# The cuproptosis-associated 11 gene signature as a predictor for outcomes and response to Bacillus Calmette-Guerin and immune checkpoint inhibitor therapies in bladder carcinoma

**DOI:** 10.3389/fimmu.2023.1126247

**Published:** 2023-05-03

**Authors:** Huiyang Yuan, Yuchen Xiu, Tiantian Liu, Yidong Fan, Dawei Xu

**Affiliations:** ^1^ Department of Urology, Qilu Hospital of Shandong University, Jinan, China; ^2^ Department of Pathology, School of Basic Medical Sciences, Shandong University, Jinan, China; ^3^ Department of Medicine, Division of Hematology, Bioclinicum and Center for Molecular Medicine, Karolinska Institute and Karolinska University Hospital Solna, Stockholm, Sweden

**Keywords:** BCG, cuproptosis, ICI, immunotherapy, prognosis, bladder carcinoma

## Abstract

Bladder cancer (BC) or carcinoma (BLCA) is predominantly derived from urothelium and includes non-muscle invasive BC (NMIBC) and muscle invasive BC (MIBC). Bacillus Calmette-Guerin (BCG) has long been applied for NMIBC to effectively reduce disease recurrence or progression, whereas immune checkpoint inhibitors (ICIs) were recently introduced to treat advanced BLCA with good efficacy. For BCG and ICI applications, reliable biomarkers are required to stratify potential responders for better personalized interventions, and ideally, they can replace or reduce invasive examinations such as cystoscopy in monitoring treatment efficacy. Here we developed the cuproptosis-associated 11 gene signature (CuAGS-11) model to accurately predict survival and response to BCG and ICI regimens in BLCA patients. In both discovery and validation cohorts where BLCA patients were divided into high- and low-risk groups based on a median CuAGS-11 score as the cutoff, the high-risk group was associated with significantly shortened overall survival (OS) and progression-free survival (PFS) independently. The survival predictive accuracy was comparable between CuAGS-11 and stage, and their combination-based nomograms showed high consistence between predicted and observed OS/PFS. The analysis of 3 BLCA cohorts treated with BCG unveiled lower response rates and higher frequencies of recurrence or progression coupled with shorter survival in CuAGS-11 high-risk groups. In contrast, almost none of patients underwent progression in low-risk groups. In IMvigor210 cohort of 298 BLCA patients treated with ICI Atezolizumab, complete/partial remissions were 3-fold higher accompanied by significantly longer OS in the CuAGS-11 low- than high-risk groups (*P* = 7.018E-06). Very similar results were obtained from the validation cohort (*P* = 8.65E-05). Further analyses of Tumor Immune Dysfunction and Exclusion (TIDE) scores revealed that CuAGS-11 high-risk groups displayed robustly higher T cell exclusion scores in both discovery (*P* = 1.96E-05) and validation *(P* = 0.008) cohorts. Collectively, the CuAGS-11 score model is a useful predictor for OS/PFS and BCG/ICI efficacy in BLCA patients. For BCG-treated patients, reduced invasive examinations are suggested for monitoring the CuAGS-11 low-risk patients. The present findings thus provide a framework to improve BLCA patient stratification for personalized interventions and to reduce invasive monitoring inspections.

## Introduction

Bladder cancers (BCs) or carcinomas (BLCAs) are the commonest urological malignancy worldwide, and up to 95% of them are originated from the urothelium (1–3). At diagnosis, the majority of BLCAs (70% – 80%) are non-muscle invasive (NMIBCs) while 20% - 30% present with muscle invasive BCs (MIBCs). NMIBCs display a high frequency of recurrence, but patients in general have a favorable outcome with long-term survival and only a small fraction (15% - 20%) advance into MIBCs (4, 5). Local and distant disseminations occur frequently in MIBCs, and many patients die from aggressive or metastatic diseases within 5 years (4, 5). During the last decades, the major clinical interventions of BLCAs largely include surgery plus intravesical Bacillus Calmette-Guerin (BCG) instillation for intermediate/high-risk NMIBCs and neoadjuvant chemotherapy for MIBCs (3, 6–9). BCG as a traditional immunotherapeutic strategy has been very successful in NMIBC treatments and this protocol is still recommended by the international guidelines as the standard care to reduce BC recurrence and progression in the present BLCA care (6–8, 10). In the recent years, promoting anti-cancer immunity by using immune checkpoint inhibitors (ICIs) as a novel strategy has been developed for clinical application and totally revolutionized the therapeutic landscape of BLCAs and other cancer types (3). By targeting PD-1/PD-L1, CTLA4, or other immune checkpoint proteins, the ICI therapy demonstrates robust efficacy in subsets of BLCA patients (3, 9). To improve stratification for better immunotherapeutic applications, numerous studies have paid great attention to biomarker identification for response to ICIs (3, 8, 11–15). Attempts to look for BCG treatment predictors are far behind, and the evaluation of BCG response depends mainly on cystoscopy, cytology and/or bladder biopsy nowadays (8), although several molecules are shown to serve as potential factors (16). In short, searching for reliable prognostic factors for patient survival, recurrence, NMIBC progression to MIBCs and treatment response are critical unmet needs, and patients with high-risk can thus be pinpointed for active surveillance and personalized intervention, thereby reducing BLCA-associated morbidity and mortality.

Copper, as an essential mineral nutrient, has long been appreciated to participate in cancer development and progression, and the copper signaling is actively involved in cancer cell proliferation, survival and metastasis (17). More recently, Tsvetkov et al. defined a copper-dependent form of regulated cell death named cuproptosis (18). During the cuproptotic process, FDX1, a reductase, and copper together bring on the lipoylation and aggregation of mitochondrial enzymes responsible for the tricarboxylic acid (TCA) cycle, and promote Fe-S cluster protein degradation, which consequently result in proteotoxic stress and eventual cell death (18). It is currently unclear whether cuproptosis plays a role in carcinogenesis or copper-mediated oncogenic function can be targeted by inducing cuproptosis. Nevertheless, recent clinical investigations have shown that cuproptosis-related factors serve as predictors for outcomes and treatment response in several cancer types (19–25). By studying patients with clear cell renal cell carcinoma (ccRCC), we showed that the cuproptosis-associated 13 gene signature (CuAGS-13) was a robust predictor for outcomes and response to ICI and targeted therapies in ccRCC (19). The association between cuproptosis and BLCA has also been explored using 10 cuproptosis factors, or cuproptosis-related genes and long non-coding RNAs (26–30). These different models consistently showed their prognostic values in outcome prediction of BLCA patients, and some of them also revealed the significant impact of cuproptosis-related factors on invasion, drug resistance, tumor microenvironments and immune cell infiltrations. However, it remains elusive whether the cuproptosis-based models are capable of predicting response to immunotherapy and survival benefits, the key clinical-related issues. Most of the above studies only examined the relationship between cuproptosis-based models and immune cell infiltrations in BLCA tumors (26, 27, 29, 30), while only in the report by Li et al, 34 BLCA patients treated with Atezolizumab (anti-PD-L1 antibody) were analyzed for their response rate using a 14 cuproptosis-related gene-containing signature (28). Unfortunately, the authors ignored the IMvigor210 cohort with 348 BLCA patients receiving Atezolizumab. The results obtained from a small cohort of 34 patients, together with lack of survival analysis, are far from the conclusive proof of the cuproptosis-related effect on ICI therapy. In addition, BCG as an immunomodulator has long been successful in NMIBC treatment as described above (7, 8), but it is currently unclear whether cuproptosis or its associated gene signature can predict BCG response. In the present study, we analyzed several BLCA cohorts and developed the BLCA-specific cuproptosis-associated 11 gene signature (CuAGS-11) as a useful predictor for outcomes and therapeutic efficacy of BCG and ICIs.

## Materials and methods

### Study workflow, data collection and processing of BLCA tumors and bladder nontumorous tissues (NTs)

**Figure 1:**
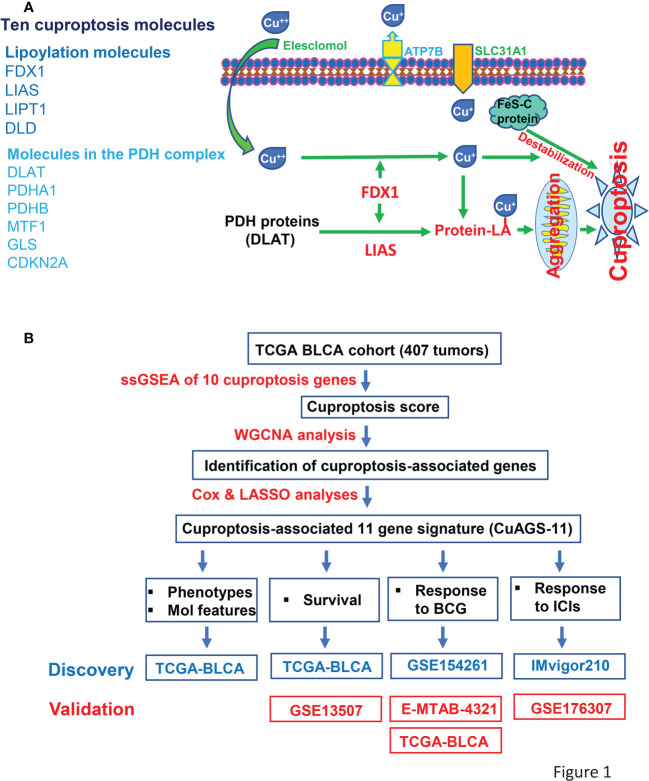
The Cuproptosis factors and study workflow. **(A)** Left panel: Ten factors involved in cuproptosis. Right panel: The cuproptosis signaling pathway. Extracellular copper Cu^++^ enters cells by binding to copper chelators and elesclomol serves as the most efficient Cu^++^ transporter. The reductase FDX1 reduces Cu^++^ to Cu^+^, a more toxic form, while lipoyl synthase (LIAS) catalyzes lipoylation of the pyruvate dehydrogenase (PDH) complex proteins including dihydrolipoamide S-acetyltransferase (DLAT) and others. Cu^+^ and lipoylation promote the protein aggregation. DLAT is one of the key enzymes participating in the tricarboxylic acid cycle, and its aggregation results in mitochondrial proteotoxic stress and subsequent cuproptotic cell death. Moreover, FDX1 and Cu^+^ induce the destabilization of Fe–S cluster proteins, further facilitating cuproptosis. Additionally, SLC31A1 and ATP7B function as the Cu^+^ importer and exporter, respectively, and regulate cuproptosis by controlling intracellular Cu^+^ concentrations. **(B)** The schematic workflow of the present study.

Based on 10 cuproptosis molecules (18) (**Figure 1A**), we sought to establish a cuproptosis-associated gene signature for BLCA prognostication using the following public databases (**Figure 1B**). (i) The TCGA cohort of BLCA (legacy) that includes 407 tumor samples and 19 bladder nontumorous tissues (31). Patient information, pathology/histology, transcriptome, mutation, and copy number variation (CNV) data were downloaded from https://gdc.cancer.gov/. Aneuploid score was from reference (32). Tumor mutation burden (TMB) was calculated using Rpackage TCGAmutations. (ii) GSE13507 (33, 34), GSE154261 (35), and GSE176307 (36) BC cohorts. The data in these cohorts were obtained from the Gene Expression Omnibus database (https://www.ncbi.nlm.nih.gov/geo/). (iii) E-MTAB-4321 cohort. The data were downloaded from https://www.ebi.ac.uk. (iv) IMvigor210 cohort. The data were from IMvigor210CoreBiologies (37, 38). For RNA sequencing data above, gene expression levels were measured using Transcripts Per Kilobase Million (TPM) and log2(x + 1) transformed. For array results (determined by 4×44K v2 microarray kit), we determined transcript abundances using probe-set values; and when multiple probes targeted the same mRNAs, the probes with largest mean values were chosen and then standardized using “Limma” package (39). During RNA sequencing and array processing, we conducted a two-step filtering. First, those genes with undetectable expression in >75% of samples were discarded. Second, we further got rid of genes with expression median absolute deviation (MAD) ≤0.01 and at the bottom 25%. The present study did not contain experimental analyses directly from human samples and animals, and thus needed no ethics permission.

### Identification of cuproptosis-associated genes whose expression correlates with 10 cuproptosis factors

**Figure 2:**
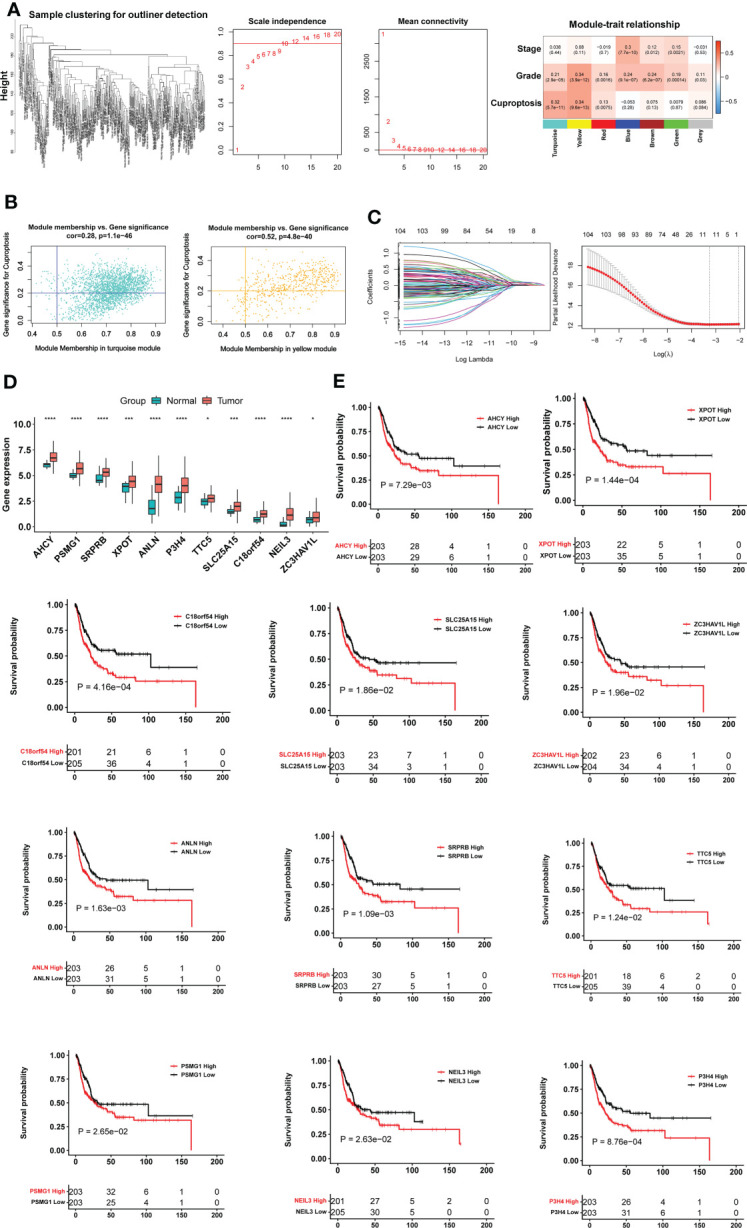
The construction of the cuproptosis-associated 11 gene signature (CuAGS-11) based on the TCGA cohort of BLCAs. **(A)** Left panel: Sample clustering trees to detect potential outliners by Pearson’s correlation in the TCGA cohort of BLCAs. Middle panel: Soft-thresholding value selection. Based on the scale-free fit index for various soft-thresholding powers (the scale independence, left panel) and mean connectivity for various soft-thresholding powers (mean connectivity, right panel), 12 was selected as a soft-threshold value (Scale-free R^2 =^ 0.90). Right panel: Gene modules correlated with cuproptosis factors as determined using Weighted gene co-expression network analysis (WGCNA). **(B)** Scatter plot of module eigengenes in the yellow (left) and turquoise (right) modules from **(A)**. The genes in the upper right are selected for further analyses. **(C)** Construction of the cuproptosis-associated 11 gene signature (CuAGS-11) for progression-free survival (PFS) prediction in BC. Left panel: LASSO coefficient profiles of the CuAGS associated with PFS. Right panel: Plots of the cross-validation error rates. Each red dot represents a lambda value with its error bar (the confidence interval for the cross-validated error rate). The analysis identified 11 cuproptosis-associated genes most relevant to PFS. **(D)** Differences in the CuAGS-11 expression between tumors and their adjacent non-tumorous tissues in the TCGA cohort of BLCA. **(E)** Kaplan–Meier survival analysis showing the impact of each gene contained in CuAGS-11 on PFS in the TCGA BLCA cohort. Patients are classified into high and low groups based on the expression of each gene in tumors using a median value as the cutoff point. *, *** and **** indicate P values <0.05, 0.001 and 0.0001, respectively.

To identify cuproptosis-associated genes, we first conducted single sample gene set enrichment analysis (ssGSEA) to obtain the normalized enrichment score (NES) according to expression levels of 10 cuproptosis genes (*FDX1*, *LIAS*, *LIPT1*, *DLD*, *DLAT*, *PDHA1*, *PDHB*, *MTF1*, *GLS* and *CDKN2A*) in the TCGA BLCA cohort (**Figure 1A**) using Rpackage “GSVA” with kcdf=Caussian and method = ssgsea. Weighted gene co-expression network analysis (WGCNA) and Pearson’s correlation were then performed to make sample clustering (tree) followed by the construction of a unsigned scale-free network, adjacency matrix and the topological overlap matrix, which eventually formed different modules (**Figure 2A**). Briefly, Pearson’s correlation was used to make sample clustering trees and none of the TCGA samples were outliners (with height >220) (**Figure 2A** left). To define the optimal soft threshold value, we set up 1:20 as a power value, and when the scale independence reached 0.9 while mean connectivity was <100, the soft threshold value 12 was obtained (**Figure 2A** middle). Based on this soft threshold setting, we constructed a unsigned scale-free network, adjacency matrix and topological overlap matrix through which the number of genes in each module was defined (maxBlockSize = 6000 and minModuleSize = 50). The function “WGCNA::blockwiseModules” was employed to assign genes into appropriate modules (**Figure 2A** right). The correlation between each module and cuproptosis ssGSEA-NES together with clinical variables (stage and grade) was then evaluated, and by setting correlation R >0.30, we acquired the yellow (R = 0.34 with 559 genes) and turquoise modules (R = 0.32 with 2525 genes) (**Figure 2A** right). Further filtering out genes at bottom Rs in these two modules (MM correlation R > 0.5, and GS correlation R > 0.2, P < 0.05) reduced the gene numbers to 392 and 1 586 in yellow and turquoise module, respectively. We then made COX and LASSO regression analyses to determine effects of these genes on patient progression-free survival (PFS) and expression differences between tumors and NTs (**Figures 2B, C**). Finally, 11 genes were obtained as the cuproptosis-associated 11 gene signature, which we named CuAGS-11. These 11 genes include C18orf54, NEIL3, ANLN, AHCY, PSMG1, TTC5, SRPRB, XPOT, ZC3HAV1L, SLC25A15 and P3H4.

### Construction of the CuAGS-11 risk score

Based on expression levels of 11 genes above, the CuAGS-11 score in each sample was calculated using the following formula:

Score = Σ βi × RNAi, where βi is the coefficient of the i-th gene in multivariable Cox regression analysis, and RNAi is RNA abundance of gene i. The obtained score values were further standardized using the scale function. Patients were classified into the high- and low-risk groups using the median score as a cut-off point. Differences in survival (OS, PFS and RFS) and BCG or ICI treatment efficacy between the CuAGS-11 high- and low-risk groups were then compared.

### Time-dependent receiver operating characteristic (ROC) curves and construction of a survival predictive nomogram

Time-dependent ROC curves and area under curves (AUCs) were used to estimate the accuracy of identified survival predictors (CuAGS-11 model and stage) in BLCA patients and made using Rpackage “timeROC”. We performed Cox regression analysis to evaluate the effect of the CuAGS-11 score and clinical parameters on survival in the TCGA and GSE13507 BLCA cohorts and established a predictive nomogram by using independent survival predictors in both cohorts to predict 1-, 3-, and 5-year survival (OS and/or PFS). The model-based predictive survival time against the observed one was plotted using calibration curves. R package “regplot” was used to make nomograms and to assess their predicative ability.

### Gene set enrichment analysis (GSEA)

Based on the median CuAGS-11 score values in BLCA cohorts, we categorized tumors into low- and high-risk groups. Reference gene signatures required for Kyoto Encyclopedia of Genes and Genomes (KEGG) and Hallmark analysis were downloaded from https://www.gsea-msigdb.org/gsea/index.jsp (h.all.v2022.1.Hs.symbols.gmt’ and ‘c2.cp.kegg.v2022.1.Hs.symbols.gmt’). Differences in KEGG and hallmark pathways between two risk groups were evaluated using GSEA (version 4.2.1). Adjusted *P* value <0.05 and *FDR <*0.25 were regarded as significantly over- or under-represented pathways.

### Analyses for proliferation, cancer stemness, and epithelial–mesenchymal transition (EMT) scores

Proliferation statuses were estimated using expression levels of Ki-67 mRNA and cell cycle scores, respectively. Cell cycle, stemness and EMT signature scores were calculated based on ssGSEA or as the median z-score of signature gene panels for each sample. These signatures are as follow: Cell Cycle: CDK2, CDK4, CDK6, BUB1B, CCNE1, POLQ, AURKA, KI-67 and CCNB2 (40). Stemness score was assessed according to the mRNA expression-based stemness developed by Malta et al. (41). EMT score was calculated based on the dbEMT signature from http://dbemt.bioinfo-minzhao.org/ (42).

### Tumor immune dysfunction and exclusion (TIDE) score analysis

TIDE score is evaluated according to myeloid-derived suppressor cell (MDSC), macrophage M2, T cell Dysfunction and Exclusion (43). TIDE score for BLCA cohorts treated with Atezolizumab was calculated online at http://tide.dfci.harvard.edu/. mRNA abundance was standardized with use of the all sample average expression as the normalization control prior to TIDE score calculations.

### Statistical analysis

All statistical analyses in the present study were conducted by using R package version 4.0.5. We performed Wilcox and K-W sum tests to determine differences between two groups and among multi groups, respectively. Spearman’s Rank-Order Correlation coefficient was used to assess correlation coefficient R between two variables. Survival analyses were carried out by using log-rank test, and Kaplan–Meier survival curves for visualization of OS, PFS and RFS were done using “Survival” and “Survminer” packages. Univariable and multivariable Cox regression analyses were employed to measure effects (HR and 95% CI) of quantitative predictive parameters on OS, PFS or RFS. *P* values < 0.05 were considered statistically significant. FDR correction was also performed to measure a statistical significance (< 0.25) when needed.

## Results

### The CuAGS-11 model establishment based on the TCGA cohort of BLCA analysis

We first evaluated 10 cuproptosis molecules as survival predictors but failed to set up a satisfied model in the TCGA BLCA cohort (data not shown) (31). By analyzing cuproptosis-correlated genes as described in Methods, we developed the cuproptosis-associated 11 gene signature (CuAGS-11). These 11 genes include C18orf54, NEIL3, ANLN, AHCY, PSMG1, TTC5, SRPRB, XPOT, ZC3HAV1L, SLC25A15 and P3H4. The expression of these 11 genes was significantly higher in BLCA tumors than in their NT counterparts (**Figure 2D**). Survival analyses unraveled the significant association of PFS with each of 11 factors when patients were categorized into high and low groups using a median expression value as the cutoff (**Figure 2E**). We then calculated CuAGS-11 score in each tumor, and divided patients into high- and low-risk groups using the median CuAGS-11 score value as a cut-off point. The CuAGS-11 score-based grouping of the TCGA BLCA tumors was significantly associated with patient age, gender, grade, stage and metastasis (**Table S1**).

### Enrichments of BLCA basal subtype and aggressive features in the CuAGS-11 high-risk tumors

**Figure 3:**
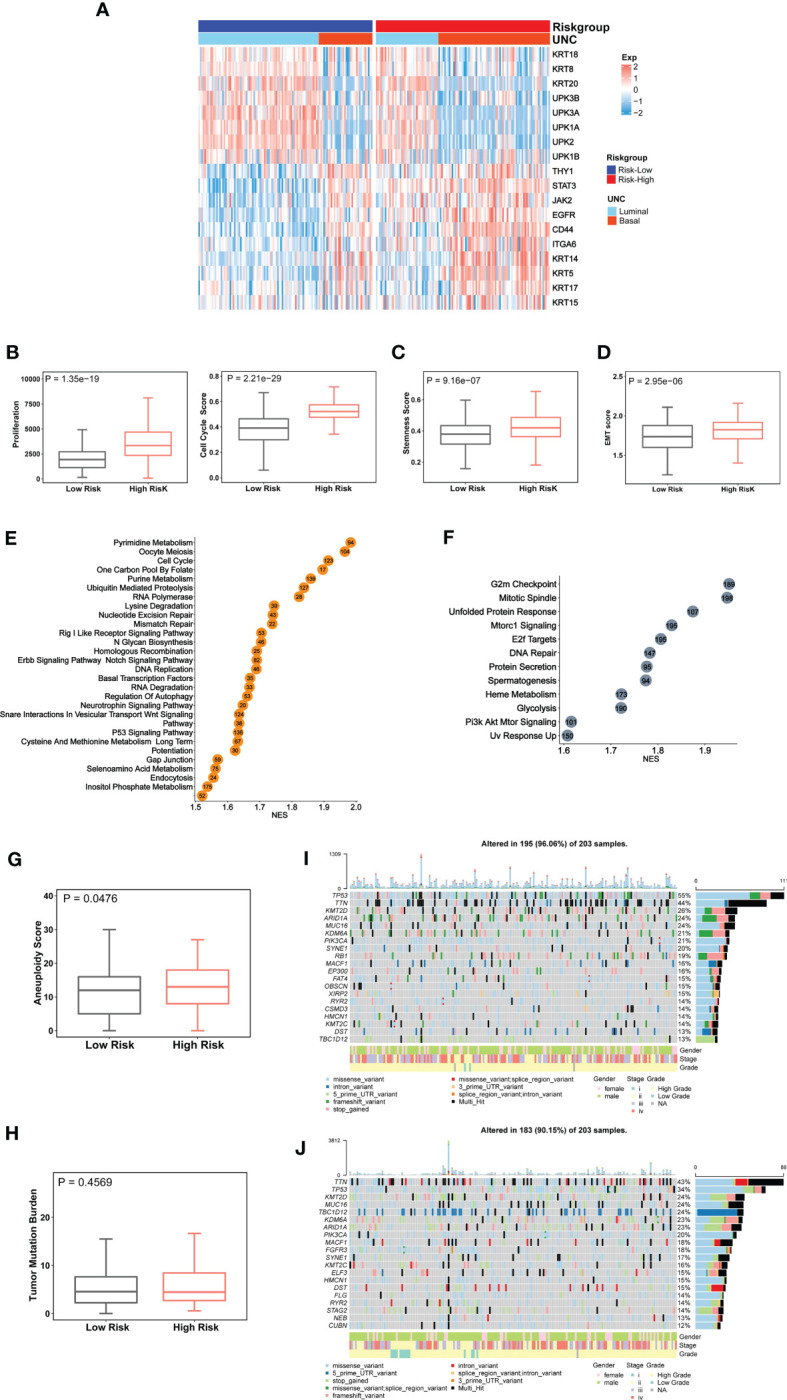
The CuAGS-11 association with molecular classification, aggressive phenotypes, signaling and genomic alterations in bladder carcinoma (BLCA). The TCGA cohort of BLCA was analyzed. Patients were divided into CuGAS-11 high- and low-risk groups using the median CuAGS-11 score value as a cut-off point. **(A)** The enriched basal subtype of BLCAs in CuAGS-11 high-risk patients. Patients were classified into luminal and basal subtypes based on their gene expression profiles shown in the figure. **(B-D)** Enhanced proliferation **(B)**, stemness **(C)** and EMT **(D)** in CuAGS-11 high-risk tumors. Proliferation was assessed using Ki-67 expression (B, left) and cell cycle scores (B, right) in BLCA tumors. Stemness and EMT evaluation was performed based on their gene expression signatures. **(C, D)** Univariable and multivariable Cox regression analyses of OS and PFS in BLCA patients, respectively. **(E, F)** The enriched oncogenic pathways in CuAGS-11 high-risk BLCA tumors. GSEA analyses were carried out to define the overrepresented KEGG pathways (left) and hallmarks (right) CuAGS-11 high-risk BLCA tumors. **(G, H)** Increased aneuploidy score **(G)** but not tumor mutation burden (TMB) **(H)** in CuAGS-11 high-risk BLCA tumors. **(I, J)** Higher frequencies of *TP53* gene alterations in CuAGS-11 high-risk BLCA tumors.

BLCAs are in general stratified into luminal and basal subtypes according to their featured gene expression signatures (44–48). The luminal subtype is overrepresented by urothelium differentiation markers and transcription factors, while the basal one is poorly differentiated (5, 44, 48). To examine a potential association between molecular and CuAGS-11 subtypes, we analyzed 233 BLCA tumors well characterized for their differentiation subtypes in the TCGA cohort. As shown in **Figure 3A**, the basal subtype was significantly enriched in the CuAGS-11 high-risk group (high- vs low-risk: *P* = 5.193E-07).

Because the basal BLCA subtype is enriched with cycling and stem- and/or mesenchymal-like cells (5, 44), we further determined proliferation, stemness and EMT markers in those tumors. For proliferation analyses, Ki-67 was first used as the specific biomarker, and the CuAGS-11 high-risk tumors expressed significantly higher levels of Ki-67 mRNA (high- vs low-risk: *P* = 1.35E-19) (**Figure 3B**). Then, cell cycle scores based on ssGSEA were evaluated and similar results were obtained (high- vs low-risk: *P* = 2.21E-29) (**Figure 3B**). BLCA stem cell and EMT phenotype analyses showed significantly higher stemness and EMT scores in CuAGS-11 high-risk tumors (high- vs low-risk: *P* = 9.16E-07 and 2.95E-06 for stemness and EMT, respectively) (**Figures 3C, D**). Consistent with these findings, the GSEA hallmark analysis revealed overrepresentation of G2M checkpoint, mitotic spindle, E2F targets, glycolysis, PIK3-AKT-MTOR signaling and among others in the CuAGS-11 high-risk tumors (**Figure 3E**). GSEA KEGG analysis showed that cell cycle, MTOR, ERBB2, basal transcription factor, TP53 and other pathways were highly enriched in the CuAGS-11 high-risk tumors (**Figure 3F**).

### Genomic alterations and their association with the CuAGS-11 model

We then probed whether there were differences in genomic alterations between the CuAGS-11 high- and low-risk tumors. First, global genomic aberrations including aneuploidy and TMB were evaluated. Aneuploidy scores were significantly higher in CuAGS-11 high- than low-risk tumors (*P* = 0.048) (**Figure 3G**), while there was no difference in TMB (**Figure 3H**). Alterations of individual genes were then compared, and we observed a significantly higher frequency of *TP53* gene aberrations in the CuAGS-11 high-risk tumors (high- vs low-risk: 55% vs 34%, *P*<0.05 and FDR<0.05) (**Figures 3I, J**).

### The CuAGS-11 score for survival prediction in BLCAs

**Figure 4:**
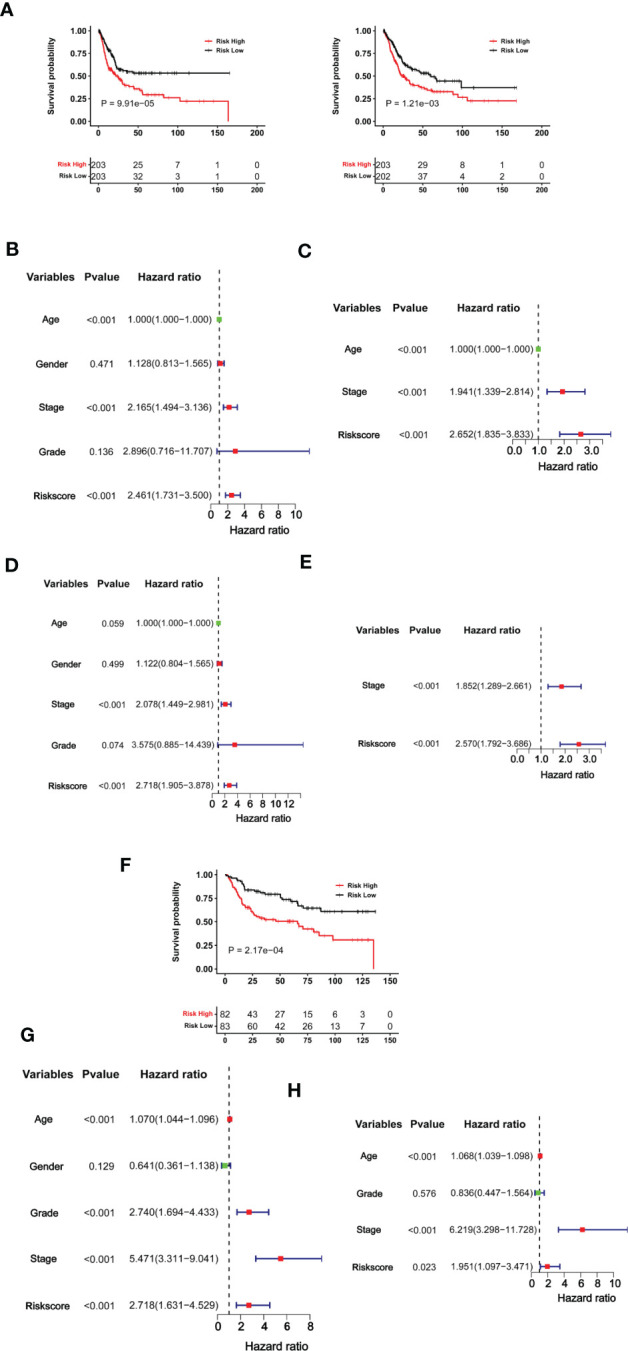
The CuAGS-11 model for BLCA survival prediction. **(A–E)** The TCGA cohort BLCA analysis. Patients were classified into high- and low-risk groups based on the CuGAS-11 score using a median value as the cutoff. **(A)** Kaplan–Meier survival analysis showing the significant association of the CuGAS-11 score with OS (left) and PFS (right) in the TCGA BLCA cohort. **(B, C)** Univariable and multivariable Cox regression analyses of OS in BLCA patients, respectively. **(D, E)** Univariable and multivariable Cox regression analyses of PFS in BLCA patients, respectively. **(F–H)** GSE13507 cohort analyses. Patients were classified into high- and low-risk groups based on the CuGAS-11 score using a median value as the cutoff. **(F)** The significant association between the CuGAS-11 high-risk group and shorter OS as shown by Kaplan–Meier survival analysis. **(G, H)** Univariable and multivariable Cox regression analyses of the CuGAS-11 effect on OS in GSE13507 cohort of BLCA patients, respectively.

Having established the CuAGS-11 score model in BLCAs, we then evaluated whether it had impacts on patient survival (OS and PFS) from the TCGA BLCA cohort as a discovery one (**Table S1**). These patients were categorized into high- and low-risk groups using the median CuAGS-11 score value as a cut-off point. Patients in the high-risk group had significantly shorter OS (*P* = 0.0001) and PFS (*P* = 0.001), as assessed by the Kaplan-Meier analysis (**Figure 4A**). Univariable COX regression OS analyses of age, gender, stage, grade, and the CuAGS-11 model showed that age (>60 yrs), advanced stages and CuAGS-11 (high-risk) were associated with significantly shorter OS (**Figure 4B**), while all these three variables remained highly significant in the multivariable COX regression analysis (**Figure 4C**). We observed a similar association between patient age (>60 yrs), advanced stages, and CuAGS-11 score (high-risk) and shorter PFS in the univariable COX regression analysis (**Figure 4D**). Multivariable COX analyses unraveled that only stage and CuAGS-11 risk score were independent predictive variables for patient PFS (**Figure 4E**).

We further determined the impact of the CuAGS-11 score on patient survival in the GSE13507 BLCA cohort to validate the findings obtained from the TCGA BLCA patients above. The GSE13507 cohort included 165 patients with BLCA (33, 34), and there were only OS data available. The patient characteristics were summarized in **Table S2**. As expected, patients in the CuAGS-11 high-risk group had significantly shorter OS (*P* = 0.0002) (**Figure 4F**). In univariable COX regression analyses, OS was significantly associated with age, grade, stage and CuAGS-11 score (**Figure 4G**), whereas age, stage and CuAGS-11 score served as independent prognostic factors, according to multivariable Cox regression analysis (**Figure 4H**).

**Figure 5:**
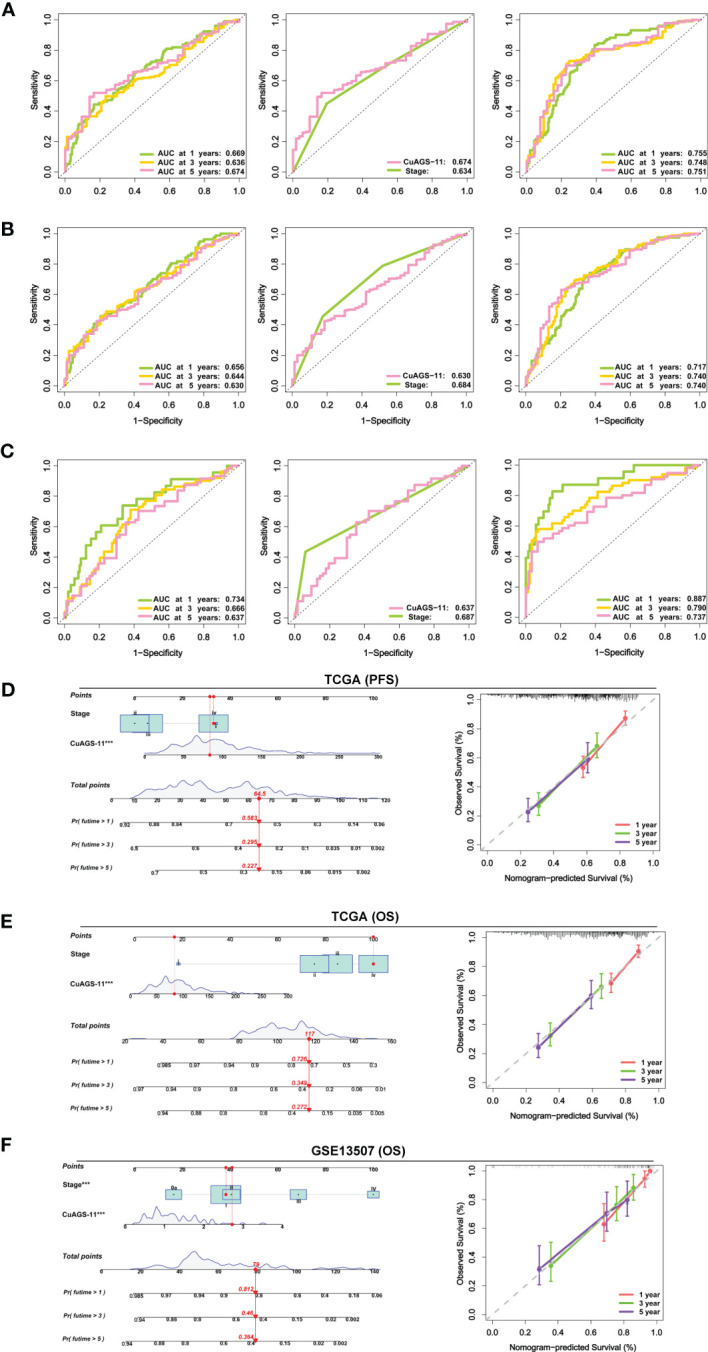
The CuAGS-11 model accuracy for survival prediction as determined by time-dependent ROC curves and nomograms. **(A)** The area under curves (AUCs) for PFS prediction using the CuAGS-11 model and/or stage in the TCGA BLCA cohort. Left: The AUCs showing the CuAGS-11 model accuracy in predicting 1-, 3- and 5-year OS. Middle: Comparison of AUCs between CuAGS-11 model and stage in predicting 5-year PFS. Right: The AUCs in predicting 1-, 3- and 5-year PFS by the combination of CuAGS-11 and stage. **(B)** The AUCs for OS prediction using the CuAGS-11 model and/or stage in the TCGA BLCA cohort. Left: The AUCs showing the CuAGS-11 model accuracy in predicting 1-, 3- and 5-year OS. Middle: Comparison of AUCs between CuAGS-11 model and stage in predicting 5-year OS. Right: The AUCs in predicting 1-, 3- and 5-year OS by the combination of CuAGS-11 and stage. **(C)** The AUCs for OS prediction using the CuAGS-11 model and/or stage in the GSE13507 BLCA cohort. Left: The OS prediction AUCs showing the CuAGS-11 model accuracy in predicting 1-, 3- and 5-year PFS. Middle: Comparison of AUCs between CuAGS-11 model and stage in predicting 5-year OS. Right: The AUCs in predicting 1-, 3- and 5-year OS by the combination of CuAGS-11 and stage. **(D–F)** The nomograms composed of CuAGS-11 model and stage for predicting 1-, 3- and 5-year PFS in TCGA **(D)**, OS in TCGA **(E)** and GSE13507 **(F)** BLCA cohorts, respectively. *** indicate P values <0.001.

We then conducted time-dependent ROC and AUC analyses to evaluate the predictive ability of the CuAGS-11 model in the TCGA and GSE13507 BLCA cohorts. For TCGA patients, AUCs for 1, 3 and 5 year PFS by the CuAGS-11 model were 0.669, 0.634 and 0.674, respectively (**Figure 5A** left panel). Like CuAGS-11 model, the stage was also an independent prognostic factor for OS and/or PFS in both cohorts, and moreover, the BLCA stage was a well-established predictor for long-term survival. Thus, we made a comparison of 5-year PFS prediction between CuAGS-11 model and stage. A slightly bigger AUC was observed for the CuAGS-11 model (**Figure 5A** middle panel). We further combined CuAGS-11 with stage together and resulting AUCs increased substantially in predicting all PFS time points (**Figure 5A** right panel). We then conducted the same analyses for OS in both TCGA and GSE13507 BLCA cohorts, and largely similar results were obtained (**Figures 5B, C**). Accordingly, we further established prognostic nomograms composed of CuAGS-11 score and stage. In the TCGA cohorts, the CuAGS-11/stage nomogram almost precisely predicted the possibility of both PFS and OS at 1, 3 and 5 years (**Figures 5D, E**). A highly accurate prediction of OS by the CuAGS-11/stage nomogram was observed in the GSE13507 cohort, too (**Figure 5F**).

### The CuAGS-11 model as a predictor for response to BCG treatment

**Figure 6:**
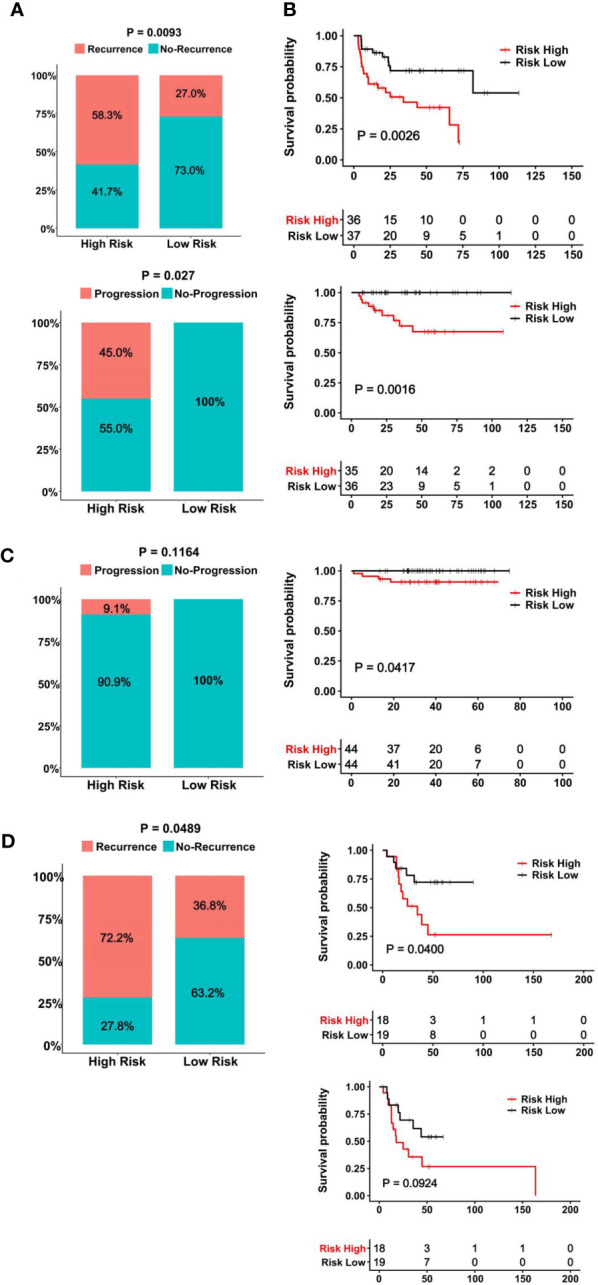
The CuAGS-11 model to predict BCG response and survival in BLCA patients. In all analyzed BLCA cohorts, patients were classified into high- and low-risk groups based on the CuGAS-11 score using a median value as the cutoff. **(A, B)** The GSE154261 cohort analyses. **(A)** Higher frequencies of recurrence (Top) and progression (Bottom) in the CuAGS-11 high-risk group patients. **(B)** Differences in RFS (Top) and PFS (Bottom) between the CuAGS-11 high- and low-risk group patients treated with BCG. **(C)** The E-MTAB-4321 cohort analyses. Left panel: All the recurred patients presented in the CuAGS-11 high-risk group. Right panel: Shorter PFS in the CuAGS-11 high-risk group patients. **(D)** The analysis of 37 BCG-treated patients with MIBC in the TCGA cohort. Left panel: Higher frequencies of recurrence in the CuAGS-11 high-risk group patients. Right panel: Differences in OS (top) and PFS (bottom) between the CuAGS-11 high- and low-risk group patients treated with BCG.

The clinic-pathological variables have been mainly used to evaluate response to BCG therapy (8). We sought to determine whether the CuAGS-11 score could serve as such a predictive biomarker. The GSE154261 BLCA cohort (35) analysis of 73 BCG-treated patients showed that the recurrence and non-recurrence rates were 58.3% and 41.7% in the CuAGS-11 high-risk group, while 27.0% and 73.0% in the low-risk one, respectively (*P* = 0.009) (**Figure 6A** top) (**Table S3**). In the low-risk group, all the patients displayed stable disease status, whereas 45% of the high-risk patients underwent progression (*P* = 0.027) (**Figure 6A** bottom). Consistently, the recurrence-free survival (RFS) and PFS were both significantly shorter in the CuAGS-11 high-risk group (*P* = 0.003 and 0.002, respectively) (**Figure 6B** top and bottom). We further analyzed the E-MTAB-4321 cohort of 88 T1 BLCA patients who received BCG therapy (49) (**Table S4**). None of 44 CuAGS-11 low-risk patients had disease progression, while 4 in the high-risk group exhibited progressive disease, although the difference was not statistically significant (*P* = 0.116) (**Figure 6C**). Nevertheless, PFS was significantly shorter in the CuAGS-11 high-risk patients (*P* = 0.042) (**Figure 6C**). Finally, 37 patients with MIBC in the TCGA cohort (31) were treated with BCG, and the recurrence rates were 72.2% and 36.8% for the CuAGS-11 high- and low-risk groups, respectively (*P* = 0.049) (**Figure 6D**) (**Table S5**). OS was significantly shorter in the CuAGS-11 high-risk group (*P* = 0.040), whereas PFS was also shorter in this group but did not reach a statistical level (*P* = 0.092) (**Figure 6E**).

### The CuAGS-11 model as a predictor for response to *Atezolizumab* therapy

**Figure 7:**
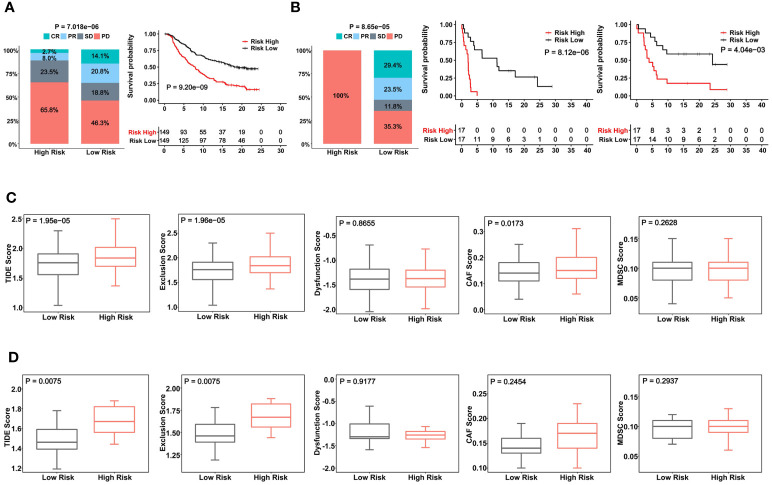
The CuAGS-11 model to predict patient response to Atezolizumab, survival and immunologically cold phenotypes in BLCA. In analyzed BLCA cohorts, patients were classified into high- and low-risk groups based on the CuGAS-11 score using a median value as the cutoff. **(A)** The CuAGS-11 model prediction of patient response to Atezolizumab (left) and OS (right) in IMvigor210 cohort. **(B)** GSE176307 cohort analyses of patient response to Atezolizumab (left) and survival (OS: middle and PFS: right) based on the CuAGS-11 model. **(C)** TIDE score analyses of IMvigor210 cohort showing robustly higher T cell exclusion score in the CuAGS-11 high-risk group patients. **(D)** TIDE score analyses of GSE176307 cohort showing robustly higher T cell exclusion score in the CuAGS-11 high-risk group patients.

ICI therapy has been applied for MIBCs with good efficacy in subsets of patients (3). We further assessed whether the CuAGS-11 score was able to predict patient response to ICIs. For this purpose, IMvigor210 and GSE176307 cohorts were analyzed as the discovery and validation sets, respectively. A total of 348 patients received Atezolizumab (anti-PD-L1 antibody) therapy and 298 of them had response information available in IMvigor210 cohorts (37, 38) (**Table S6**). These 298 patients were divided into the CuAGS-11 high- and low-risk groups based on the median score value. Patient responses to Atezolizumab were categorized into complete remission (CR), partial remission (PR), stable disease (SD) and progressive disease (PD), respectively (38). CR, PR, SD and PD were 2.7%, 8.0%, 23.5% and 65.8% in the high-risk group, whereas 14.1%, 20.8%, 18.8% and 46.3% in the low-risk group, respectively (*P* = 7.018E-06) (**Figure 7A**). OS information was available in this cohort and Kaplan-Meier analysis showed a dramatically shortened OS in the CuAGS-11 high-risk group (*P* = 9.20E-09) (**Figure 7A**). The GSE176307 cohort of 34 BLCA patients treated with Atezolizumab (36) was then analyzed for validation (**Table S7**). All 17 patients in the high-risk group underwent disease progression, while more than half of patients in the low-risk group acquired CR (29.4%) or PR (23.5%) and only 35.3% of them had BC progression (high- vs low-risk, *P* = 8.65E-05) (**Figure 7B**). In accordance with their response rates, significantly longer OS and PFS were observed in the CuAGS-11 low-risk group (*P* = 8.12E-06 and 4.04E-03 for OS and PFS, respectively) (**Figure 7B**).

Given these observations, we further probed potential differences in infiltrated stroma and immune cells between the CuAGS-11 high- and low-risk tumors. To this end, we compared their TIDE scores. TIDE has been shown to predict ICI responses and determine mechanisms underlying tumor immune evasion (43). In the IMvigor210 cohort, the CuAGS-11 high-risk tumors displayed significantly higher TIDE score than did the low-risk tumors (*P* = 1.95E-05), and more specifically, robustly higher T cell exclusion score was observed in CuAGS-11 high-risk tumors (high- vs low-risk: *P* = 1.96E-05) (**Figure 7C**). The GSE176307 cohort analysis showed similar score differences in TIDE (*P =* 0.008*)* and T cell exclusion (*P =* 0.008*)* between high- and low-risk groups (**Figure 7D**).

## Discussion

BLCAs derived from urothelial cells are heterogenous and include NMIBCs and MIBCs with different outcomes and different clinical interventions (4, 5). Reliable biomarkers are highly required to stratify patient risk and to tailor treatment regimens (3). Based on the cuproptosis-associated gene analysis, we develop the CuAGS-11 model for such a purpose. Our results presented herein demonstrate that CuAGS-11 serves as a useful predictor for BLCA patient survival and response to immunotherapies including BCG and ICIs.

Copper has long been appreciated to participate in oncogenesis (17). Cuproptosis, a newly identified form of regulated cell death (RCD), is copper-dependent cell death caused by FDX1-mediated mitochondrial protein lipoylation (18). It is currently unclear whether cuproptosis, like apoptosis or other types of RCD, plays any parts in carcinogenesis. We recently developed a CuAGS-13 model to accurately predict ccRCC outcomes and patient response to targeted and ICI therapies (19), however, this same model failed to do so in BLCAs (data not shown). Intriguingly, the direct application of 10 cuproptosis factors did not show any prognostic values in either BLCAs or ccRCCs. Moreover, the genes in the CuAGS-11 model are totally different from those in the CuAGS-13 (19) or from any other cuproptosis-related models. Thus, cuproptosis-based models are context-dependent and their prognostic powers very significantly. In the present study, the CuAGS-11 model is constructed based on 10 cuproptosis factors, but the scores are strongly correlated with many key aggressive characteristics in BLCAs in positive manners. Thus, the CuAGS-11 model represents a classifier integrated with pathological/molecular and many other BLCA features. Despite such broad connections, the CuAGS-11 score predicts BLCA patient survival independently of any other variables.

BCG, a live attenuated strain of Mycobacterium bovis, was first applied for patients with NMIBC in 1976 (10), and since then, intravesical BCG therapy has been the most effective treatment strategy to prevent recurrence for intermediate- and high-risk NMIBCs (8). If without adjuvant therapies, recurrence occurs in up to 70% of patients who received tumor resection (8). Mechanisms underlying BCG against BLCAs remain incompletely understood, but accumulated studies indicate that BCG as an immunomodulator stimulates both innate and acquired immune responses, thereby exerting a therapeutic efficacy (8, 50). Li et al. recently showed that BCG treatment failure was associated with enhanced PD-L1 and FGFBP1 expression (51). In addition, BCG may have direct impacts on BLCA cells. Despite tremendous advances in next generation sequencing and other high-throughput technologies, there is still lack of reliable molecular predictors for response to BCG. Clinic-pathological variables combined with cystoscopy have so far served as major approaches to predict and measure potential response to BCG (7, 8). In clinical practice nowadays, cystoscopy, cytology and/or bladder biopsy are used to determine response at 3 months and 6 months following a BCG induction regimen (7, 8). Cystoscope examination is invasive and costly. In the present investigations, we showed a high accuracy of the CuAGS-11 model in prognosticating BCG responders. Based on our analyses of 3 BLCA cohorts treated with BCG, almost all patients with disease progression were observed in the CuAGS-11 high-risk group. For T1 patients, no recurrence occurred in the CuAGS-11 low-risk group. These proof-of-concept results suggest that the invasive examination may not be required or at least reduced for CuAGS-11 low-risk patients, which is worthy of further studies.

In the recent years, the ICI therapy has been applied for advanced cancers including MIBCs (3). As only subsets of MIBCs respond to ICIs, distinguishing potential responders from non-responders should greatly contribute to personalized application of ICIs and the development of accurate predictive biomarkers is thus critical. Here we found that the CuAGS-11 model was similarly useful for stratifying ICI responders in 2 cohort patients treated with Atezolizumab. In the IMvigor210 cohort, the CR/PR rate was > 3-fold higher in the low- than high-risk group. The GSE176307 cohort analysis showed that all 17 patients underwent progression in the CuAGS-11 high-risk group, whereas 9 of 17 (54.7%) low-risk patients obtained CR or PR. Consistently, patients in the low-risk group had significantly longer PFS and/or OS. TMB has been shown as a reliable predictor for response to ICI therapy in BLCAs (11), but the CuAGS-11 signature was not related to TMB. TIDE analyses of these two cohorts consistently showed that CuAGS-11 high-risk tumors were characterized by T cell exclusion, which suggests that the CuAGS-11 model can help identify BC tumors with an immunologically cold phenotype. Poor response to ICIs is thus conceivable in patients with CuAGS-11 high tumors. It is currently unclear what is a mechanistic link between CuAGS-11 score and T cell exclusion in BLCAs and whether cuproptosis is involved in immune cell fate decision or T cell-mediated tumor destruction. Elucidating these important issues should contribute to improvement of BLCA immunotherapy.

In conclusion, we constructed the CuAGS-11 score model for prediction of survival and response to BCG and ICI therapies in BLCAs. This model, although derived from cuproptosis-associated factors, is a classifier integrated with molecular and other features of BLCA. Importantly, for patients receiving BCG, recurrence occurs predominantly in the CuAGS-11 high-risk group, and no disease progression was observed in the low-risk patients. Thus, it may be unnecessary to monitor the CuAGS-11 low-risk patients using invasive examinations routinely. The present findings further show that the CuAGS-11 model is helpful to identify BLCA tumors with an immunologically cold phenotype and to distinguish between ICI responders and non-responders. Taken together, the CuAGS-11 score model may significantly improve BLCA patient stratification for tailored patient interventions, reducing BLCA-associated morbidity and mortality. It is worth validating these observations in clinical practices.

## Data availability statement

Source data downloaded from public databases are provided with this paper. Any additional information required to reanalyze the data reported in this paper is available from the corresponding authors upon reasonable request.

## Author contributions

HY, TL, YF, and DX designed the study. HY and YX performed bioinformatic analysis. YF and DX supervised the study. HY, TL, YF, and DX wrote and revised the manuscript. All authors reviewed and edited the manuscript. All authors contributed to the article and approved the submitted version.
