# Supplementary material for: The cuproptosis-associated 11 gene signature as a predictor for outcomes and response to Bacillus Calmette-Guerin and immune checkpoint inhibitor therapies in bladder carcinoma
Source: Front Immunol. 2023 May 3;14:1126247. doi: 10.3389/fimmu.2023.1126247 (PMC10189141; doi:10.3389/fimmu.2023.1126247)
Supplement: Supplementary file 1 [file DataSheet_1.pdf]

The Cuproptosis-associated 11 gene signature as a predictor for outcomes and response to Bacillus Calmette-Guerin and immune checkpoint inhibitor therapies in bladder cancer

Huiyang Yuan[1], Yuchen Xiu[2], Tiantian Liu[2], Yidong Fan[1] and Dawei Xu[3]

Supplementary materials include Table S1-S7.

Table S1. Clinic-pathological characteristics of the TCGA BC cohort and association with the CuAGS-11 risk group (Low vs High)

	Risk-Low	Risk-High	P
	N = 203	***N = 203***	
**Age (yrs, mean ± SEM[a])**	68.7 (11.2)	68.4 (9.99)	0.758
**Sex (N, %)**			0.429
Male	154 (75.9%)	146 (71.9%)	
Female	49 (24.1%)	57 (28.1%)	
**T stage (N, %)**			0.024
T1	2 (1.06%)	1 (0.55%)	
T2	69 (36.5%)	49 (26.8%)	
T3	84 (44.4%)	109 (59.6%)	
T4	34 (18.0%)	24 (13.1%)	
**Lymph node metastasis (N, %)**			0.217
N0	123 (66.5%)	112 (62.9%)	
N1	17 (9.19%)	29 (16.3%)	
N2	40 (21.6%)	34 (19.1%)	
N3	5 (2.70%)	3 (1.69%)	
**Metastasis (N, %)**			0.393
M0	106 (96.4%)	89 (92.7%)	
M1	4 (3.64%)	7 (7.29%)	
**AJCC stage[b] (N, %)**			0.232
I	2 (0.99%)	0 (0.00%)	
II	71 (35.1%)	57 (28.4%)	
III	66 (32.7%)	74 (36.8%)	
IV	63 (31.2%)	70 (34.8%)	
**Grade (N, %)**			0.003
Low Grade	17 (8.42%)	3 (1.49%)	
High Grade	185 (91.6%)	198 (98.5%)	

[a]SEM, standard error of mean.

[b]Tumor AJCC stages according to the American Joint Committee on Cancer (AJCC) 7th edition.

Table S2. Clinic-pathological characteristics of the GSE13507 BC cohort and association with the CuAGS-11 risk group (Low vs High)

	Risk-Low	Risk-High	P
	N = 83	***N = 82***	
**Age (yrs, mean ± SEM[a])**	64.9 (12.2)	65.5 (11.8)	0.755
**Sex (N, %)**			1.000
Female	15 (18.1%)	15 (18.3%)	
Male	68 (81.9%)	67 (81.7%)	
**T stage (N, %)**			0.594
Ta	13 (15.7%)	11 (13.4%)	
T1	42 (50.6%)	38 (46.3%)	
T2	17 (20.5%)	14 (17.1%)	
T3	7 (8.43%)	12 (14.6%)	
T4	4 (4.82%)	7 (8.54%)	
**Lymph node metastasis (N, %)**			0.377
N0	78 (94.0%)	71 (87.7%)	
N1	2 (2.41%)	6 (7.41%)	
N2	3 (3.61%)	3 (3.70%)	
N3	0 (0.00%)	1 (1.23%)	
**Metastasis (N, %)**			0.277
M0	81 (97.6%)	77 (93.9%)	
M1	2 (2.41%)	5 (6.10%)	
**Grade (N, %)**			0.233
Low Grade	57 (68.7%)	48 (58.5%)	
High Grade	26 (31.3%)	34 (41.5%)	

[a]SEM, standard error of mean.

Table S3. Clinic-pathological characteristics of the GSE154261 BC cohort of patients treated with BCG and association with the CuAGS-11 risk group (Low vs High)

	**Risk-Low**	**Risk-High**	**P**
	N = 37	***N = 36***	
**Histology**			1
NMIBC	37 (100%)	36 (100%)	
**T stage (N, %)**			1
T1	37 (100%)	36 (100%)	
**Grade (N, %)**			1
High Grade	37 (100%)	36 (100%)	
**Recurrence (N, %)**			0.014
No-Recurrence	27 (73.0%)	15 (41.7%)	
Recurrence	10 (27.0%)	21 (58.3%)	
**Progression to T2 (N, %)**			0.001
No-Progression	37 (100%)	27 (75.0%)	
Progression	0 (0.00%)	9 (25.0%)	

Table S4. Clinic-pathological characteristics of the E-MTAB-4321 BC cohort treated with BCG and association with the CuAGS-11 risk group (Low vs High)

	**Risk-Low**	**Risk-High**	**P**
	N = 44	***N = 44***	
**Age** (yrs, mean ± SEM[a])	68.0 (8.14)	69.5 (9.77)	0.445
**Sex (N, %)**			0.186
Female	6 (13.6%)	12 (27.3%)	
Male	38 (86.4%)	32 (72.7%)	
**T stage (N, %)**			0.157
Cis	1 (2.27%)	1 (2.27%)	
T1	14 (31.8%)	22 (50.0%)	
Ta	29 (65.9%)	21 (47.7%)	
**Grade (N, %)**			0.001
High grade	15 (34.1%)	32 (72.7%)	
Low grade	29 (65.9%)	12 (27.3%)	
**Tumor size (cm) (N, %)**			0.751
< 3	30 (85.7%)	28 (80.0%)	
≥ 3	5 (14.3%)	7 (20.0%)	≥ 3

[a]SEM, standard error of mean.

Table S5. Clinic-pathological characteristics of 37 patients treated with BCG in the TCGA BC cohort and association with the CuAGS-11 risk group (Low vs High)

	**Risk-Low**	**Risk-High**	**P**
	N = 19	***N = 18***	
**Age** (yrs, mean ± SEM[a])	74.0 (8.67)	66.4 (10.6)	0.024
**Sex (N, %)**			0.660
Male	15 (78.9%)	16 (88.9%)	
Female	4 (21.1%)	2 (11.1%)	
**Grade (N, %)**			1
High Grade	19 (100%)	18 (100%)	
**Recurrence (N, %)**			0.048
No-Recurrence	12 (63.2%)	5 (27.8%)	
Recurrence	7 (36.8%)	13 (72.2%)	

[a]SEM, standard error of mean.

Table S6. Clinic-pathological characteristics of the IMvigor210 BC cohort treated with Atezolizumab and association with the CuAGS-11 risk group (Low vs High)

	Risk-Low	Risk-High	P
	N = 149	***N = 149***	
**Sex (N, %)**			0.779
Female	34 (22.8%)	31 (20.8%)	
Male	115 (77.2%)	118 (79.2%)	
**ECOG mean** (SEM[a])	0.57 (0.57)	0.70 (0.54)	0.049
**Immune phenotype (N, %)**			0.001
Desert	26 (20.8%)	43 (36.1%)	
Excluded	56 (44.8%)	57 (47.9%)	
Inflamed	43 (34.4%)	19 (16.0%)	
**Response[b] (N, %)**			<0.001
CR	21 (14.1%)	4 (2.68%)	
PR	31 (20.8%)	12 (8.05%)	
SD	28 (18.8%)	35 (23.5%)	
PD	69 (46.3%)	98 (65.8%)	

[a]SEM, standard error of mean.

[b]Response:

CR, complete response;

PR, partial response;

SD, stable disease;

PD, progressive disease.

Table S7. Clinic-pathological characteristics of the GSE176307 BC cohort treated with Atezolizumab and association with the CuAGS-11 risk group (Low vs High)

	Risk-Low	Risk-High	P
	N = 17	***N = 17***	
**Sex (N, %)**			1.000
Female	7 (41.2%)	6 (35.3%)	
Male	10 (58.8%)	11 (64.7%)	
**ECOG (N, %)**			0.057
0	2 (15.4%)	7 (53.8%)	
1	6 (46.2%)	5 (38.5%)	
2	4 (30.8%)	0 (0.00%)	
3	1 (7.69%)	1 (7.69%)	
**Response[a] (N, %)**			<0.001
CR	5 (29.4%)	0 (0.00%)	
PR	4 (23.5%)	0 (0.00%)	
SD	2 (11.8%)	0 (0.00%)	
PD	6 (35.3%)	17 (100%)	

[a]Response:

CR, complete response;

PR, partial response;

SD, stable disease;

PD, progressive disease.
